# First characterization of *Plasmodium vivax* liver stage antigen (PvLSA) using synthetic peptides

**DOI:** 10.1186/1756-3305-7-64

**Published:** 2014-02-12

**Authors:** Youn-Kyoung Goo, Eun-Jeong Seo, Yeon-kyung Choi, Hyun-Il Shin, Jetsumon Sattabongkot, So-Young Ji, Chom-Kyu Chong, Shin-Hyung Cho, Won-Ja Lee, Jung-Yeon Kim

**Affiliations:** 1 Division of Malaria and Parasitic Diseases, National Institute of Health, Korea CDC, Osong Saeng-myeong 2 ro, Osong Health Technology Administration Complex 187, Osong, Republic of Korea; 2 Department of Parasitology and Tropical Medicine, Kyungpook National University School of Medicine, Daegu, Republic of Korea; 3 Department of Entomology, Armed Forces Research Institute of Medical Sciences, Bangkok, Thailand; 4 GenBody Inc., Biotech Business IC, Dankook University, Cheonan, Chungnam, 330-714, Republic of Korea

**Keywords:** *Plasmodium vivax*, Liver stage antigen, Peptides

## Abstract

**Background:**

*Plasmodium vivax* is the most widespread human malaria in tropical and subtropical countries, including the Republic of Korea. Vivax malaria is characterized by hypnozoite relapse and long latency infection by the retained liver stage of *P. vivax*, and somewhat surprisingly, little is known of the liver stage antigens of this parasite. Here, we report for the first time the characterization of a liver stage antigen of *P. vivax* (PvLSA).

**Methods:**

Five peptides located inside PvLSA were synthesized, and specific anti-sera to the respective peptides were used to localize PvLSA on *P. vivax* parasites in human liver cells by immunofluorescence. Western blotting and enzyme-linked immunosorbent assay were performed using the five peptides and sera collected from vivax malaria patients and from normal healthy controls.

**Results:**

PvLSA was localized on *P. vivax* parasites in human liver cells. Vivax malaria-infected patients were detected using the five peptides by western blotting. Furthermore, the peptides reacted with the sera of vivax malaria patients.

**Conclusions:**

These results suggest that PvLSA may function during the liver stage of *P. vivax*.

## Background

*Plasmodium vivax* is the most widespread human malaria, and afflicts several hundred million people annually. It is endemic to tropical and subtropical countries of the Americas, Africa, and Asia, including the Republic of Korea (ROK) [1-3]. Unlike *P. falciparum*, *P. vivax* is characterized by hypnozoite relapse in the liver. After being bitten by a *P. vivax*-infected mosquito, sporozoites enter hepatocytes, where most develop into schizonts that result in primary illness. However, some remain as hypnozoites, which can become active months or even years later, and cause relapse after resolution of the primary illness. Several factors have been suggested to lead to hypnozoite development, for example, a cold ambient temperature, the number of infecting sporozoites, the specific strain of the mosquito vector or *P. vivax*[4-6]. However, the mechanisms responsible for hypnozoite development and their activation are not known.

A vaccine and a diagnostic method based on antigens specific to the liver stage of *P. vivax* are needed in order to control vivax malaria, since asymptomatic carriers in latency contribute to disease transmission. In falciparum malaria, a recombinant anti-sporozoite subunit vaccine (RTS,S/AS01) targeting circumsporozoite protein (CSP) has shown best performance among vaccines developed to date, though Phase III trials are ongoing [7]. In addition, the detection of human carriers in the latent stage caused by hypnozoites is important in many countries, including the ROK, where the control strategy for vivax malaria is moving from intervention toward elimination. Therefore, an understanding of molecules specific for the liver stage could help overcome the challenge posed by vivax malaria in the setting of disease elimination.

In *P. falciparum*, liver stage antigen-3 (LSA-3) is a novel antigen expressed at the pre-erythrocytic stage [8]. A number of studies have demonstrated the potential of LSA-3 as a vaccine and serodiagnosis candidate. B- and T-cell epitopes have been characterized in LSA-3 [9], and LSA-3 antigenicity has been demonstrated in several immuno-epidemiological studies conducted in *P. falciparum* malaria-exposed populations [10]. Moreover, an enzyme-linked immunosorbent assay (ELISA) based on recombinant LSA-3 has been developed as a serodiagnostic test for *P. falciparum* in Myanmar [11]. On the other hand, little is known about the molecular characteristics of the liver stage of *P. vivax*, and the majority of studies conducted, since Garnham identified the pre-erythrocytic stage of *P. vivax* in human liver in 1947 [12], focused on the biology of hypnozoites.

Synthetic peptides derived from antigens of *Plasmodium* spp. provide practical advantages for vaccine development [13], evaluations of antigenicity [14,15], and surveys of immunologic profiles in malaria-exposed populations [16]. Furthermore, ELISA tests developed for peptides of some promising antigens now have improved performances [17].

Therefore, we synthesized peptides that span all liver stage antigens of *P. vivax* (PvLSA), and evaluated the antigenicities of these peptides by Western blotting. Finally, the efficacies of ELISA for these peptides were determined based on its ability to detect blood samples from vivax malaria patients.

## Methods

### Ethics statements

The study was performed in the ROK and in Thailand, and was approved by the ethics committee of the Korean National Institute of Health (Approval number: 2009-01CON-01-4R). An approval form was used to obtain written informed consent from each participant. In addition, all participants provided permission for the sampling of 5 ml of blood.

### Blood samples

Blood samples, which were collected in EDTA tubes, were obtained from 65 patients diagnosed with vivax malaria at local health centers (Gang-wha, Paju, Gimpo) from March to August. Microscopic examinations of Giemsa-stained thick and thin blood films were used to confirm diagnoses. Samples were also obtained from 10 asymptomatic and aparasitemic healthy volunteers confirmed as being *P. vivax* negative by microscopic examination and nested-PCR.

### Selection and synthesis of antigenic peptides on *Plasmodium vivax* liver stage antigen

**Figure 1:**
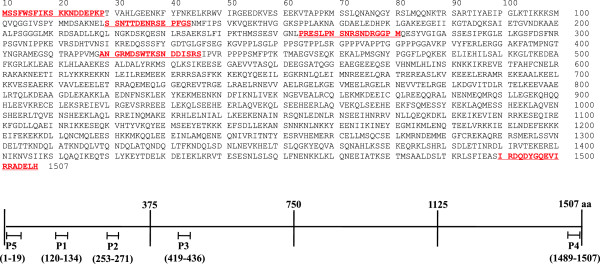
**Amino acid sequences of PvLSA and a schematic diagram of PvLSA peptides.** Selected peptides of *P. vivax* LSA, identified using bioinformatic software packages, are underlined (P1-5, P = peptide).

Here we used the liver stage antigen gene of the *P. vivax* Sal-1 strain (PvLSA; Accession No. XP_001615328). The open reading frame (ORF) of PvLSA was identified from a cDNA library of *Plasmodium vivax* Korean isolates, and 4,521 nucleotides were predicted to encode a polypeptide consisting of 1,507 amino acid residues. The molecular weight of the mature protein was 173.4 kDa, as calculated by Statistical Analysis of Protein Sequences (SAPS, https://www.ebi.ac.uk/Tools/seqstats/saps/). To select peptide candidates with high antigenicity, we used the following B-cell epitope mapping programs: BepiPred, which is based on propensity scale methods ( http://www.cbs.dtu.dk/services/BepiPred/) [18] and BCPreds, which is based on machine learning methods ( http://ailab.cs.iastate.edu/bcpreds/) [19]. These programs revealed five peptides, P1-5 (Figure 1), which were subsequently synthesized either without or with ovalbumin conjugates by Peptron Inc. (ROK).

### *Plasmodium vivax* sporozoite preparation

*P. vivax* sporozoites were prepared at the Armed Forces Research Institute of Medical Sciences (AFRIMS; Thailand), as described previously [20,21]. Briefly, sporozoites were collected from the salivary glands of *Anopheles dirus* (Bangkok Colony) mosquitoes fed the blood of vivax malaria patients. Sporozoites, in an aseptic solution containing 200 U/ml penicillin and 200 μg/ml streptomycin, were centrifuged and counted. Subsequently, they were inoculated into HC-04 cells (a human hepatocyte cell line) that had been cultured in complete medium (MEM: Ham’s F12 Gibco BRL, 1:1 v/v) supplemented with 10% fetal bovine serum (Gibco BRL), 100 U/ml penicillin, and 100 μg/ml streptomycin at 37°C for 48 h. HC-04 cells were harvested on days 1, 2, and 3 after sporozoite inoculation. 3After washing, cells were spread as a monolayer on cytospin slides (ThermoShandon, USA).

### Immunofluorescence assay (IFA)

To determine whether PvLSA was expressed in liver stage parasites, we performed an immunofluorescence assay (IFA) using HC-04 cells and specific anti-sera to the five peptides at the Armed Forces Research Institute of Medical Sciences (AFRIMS; Thailand). Anti-sera specific to the respective peptides (P1-5) were purchased from Peptron Inc. (ROK). The slides prepared as described above were first fixed with cold acetone for IFA. Next, anti-P1, P2, P3, P4, and P5 rabbit sera, and the monoclonal antibody of the circumsporozoite protein type VK210 were used at concentrations of 10 μg/ml for IFA staining. Secondary antibodies were fluoresced using isothiocyanate-conjugated anti-rabbit and human IgG (Invitrogen, USA), and fluorescence was visualized by confocal microscopy (Leica, Germany). An anti-serum to the circumsporozoite antigen expressed in *P. vivax* parasites during the early liver stage was used as a positive control.

### Sodium dodecyl sulfate-polyacrylamide gel electrophoresis (SDS-PAGE) and Western blotting

Briefly, the five ovalbumin-conjugated peptides were separated by SDS-PAGE and stained with Coomassie Blue. Separated peptide fractions were electroblotted onto Immobilon-P Transfer membranes (Millipore, USA), which were then blocked with 5% skim milk (Wako, Japan). Subsequently, membranes were probed overnight with vivax malaria sera samples diluted in 5% skim milk. Bound antibodies were reacted with horseradish peroxidase-conjugated secondary antibodies and detected using the West-Q Chemiluminescent Substrate Kit (GenDEPOT, USA).

### Enzyme-linked immunosorbent assay (ELISA)

The 60 blood samples from vivax malaria patients and 10 samples from healthy controls were subjected to ELISA as previously described with modifications [22]. Peptides without ovalbumin (P1, 2, 3, and 5) (500 ng) were coated onto 96-well microplates (Nunc, Denmark) overnight at 4°C, and then incubated with respective blood samples at a dilution of 1:200 (peptide 4, which showed weak reaction in a localization study was excluded). Second antibody binding was detected using horseradish peroxidase-conjugated anti-human IgG (Bethyl Laboratories, Inc., USA) (1:5000) and TMV (Sigma-Aldrich, USA). Optical densities were measured at 450 nm. The cut-off value of each peptide was calculated by adding 3 times the standard deviation of 10 blood samples from healthy people to the mean OD value. Samples with an OD value higher than the appropriate cut-off value were considered vivax malaria positive.

## Results and discussion

**Figure 2:**
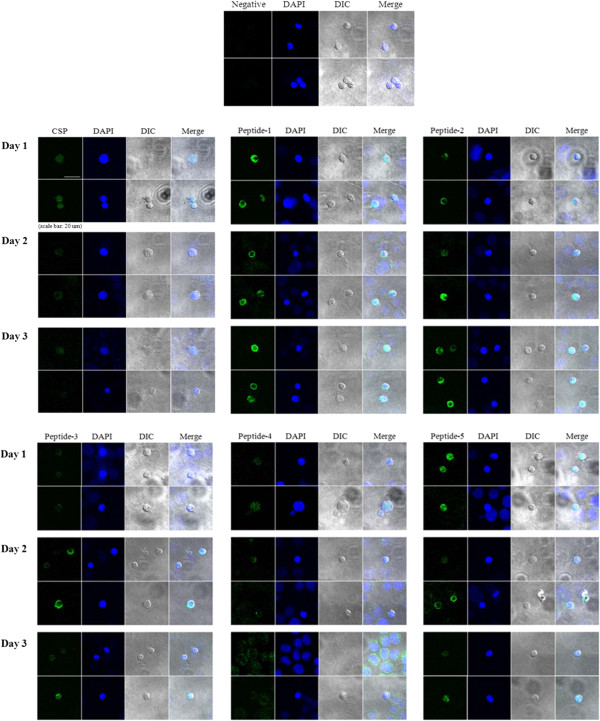
**Immunolocalization of PvLSA in *P. vivax *sporozoites.** Specific anti-sera to P1-5 and CSP (circumsporozoite protein) reacted with *P. vivax* sporozoites in hepatocytes from day 1 to day 3 post infection. (Green, peptides 1–5 and CSP; blue, DAPI)

Five antigenic peptides in a liver stage antigen of *P. vivax* (PvLSA) were predicted by two bioinformatic programs (Figure 1), and then synthesized with or without ovalbumin conjugates. These PvLSA peptides of Sal-I strain appeared to be conserved among the PvLSAs of other strains, including Brazil I, India VII, Mauritania I, North Korean, and South Korean strains according to the determination of single nucleotide polymorphisms (SNPs) of PvLSAs in PlasmoDB. Total 32 SNPs were found in PvLSAs of these six strains, and 17 of the 32 were non-synonymous SNPs; the non-synonymous/synonymous ratio was 1.13, meaning that PvLSA is a genetically diverse protein. However, 3 SNPs and other 29 SNPs were located in intron and other non-synthesized regions, respectively (Additional file 1: Figure S 1). Thus, the selected peptides were considered conserved among *P. vivax* strains. As shown in Figure 2, the purchased specific anti-sera for P 1–5 reacted with *P. vivax* parasites in the liver stage, and reactions between specific anti-sera and the parasites in human liver cells were even stronger than those between specific anti-sera and CSP; anti-serum to P4 showed relatively weak reactions as compared with those of other peptides. Reactions between specific anti-sera to peptides and *P. vivax* parasites in liver cells continued from day 1 to day 3 post-parasite infection, indicating that the five peptides spanning PvLSA are translated in the liver stage of *P. vivax* and retained during the early liver stage. Although longer culture of HC-04 cells with *P. vivax* is needed to determine whether reactions continued to the latent liver stage, these localization results demonstrate the first identification of a liver stage antigen of *P. vivax*.

**Figure 3:**
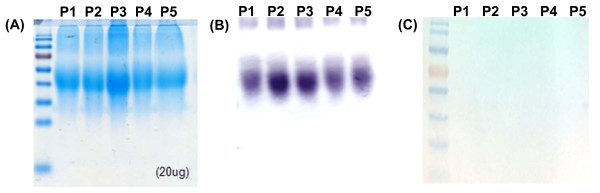
**Antigenicity of peptides 1–5 by Western blotting. (A)** Ovalbumin-conjugated peptides (P1-5) were separated by SDS-PAGE. **(B)** Sera from vivax malaria patients reacted with ovalbumin-conjugated peptides 1–5. **(C)** No reaction was observed between the ovalbumin-conjugated peptides 1–5 probed with the sera of healthy individuals.

Because LSA-3 of *P. falciparum* (PfLSA-3) is a promising candidate for a vaccine and for the serodiagnosis of falciparum malaria, we hypothesized that the five synthesized small peptides were antigenic, and thus, could be used to detect antibodies specific to PvLSA in blood samples from malaria-infected individuals. To test this hypothesis, we performed western blotting using these five peptides and blood samples obtained from vivax malaria patients exhibiting clinical symptoms and diagnosed as having vivax malaria by microscopic examination at health centers in the ROK. As shown in Figure 3, strong reactions between P1-5 and blood samples from vivax patients were observed (B), whereas no reaction was observed in the blood samples of healthy controls (C). As expected, blood samples of vivax malaria patients showed no bands when only ovalbumin was loaded (data not shown). These results indicate that the five peptides are antigenic.

Next, in order to analyze the antibody responses to PvLSA peptides in naturally infected individuals, ELISAs with the peptides were conducted using sera from 60 patients infected with *P. vivax*. As shown in Table 1, the peptides spanning PvLSA reacted with blood samples from patients of vivax malaria. In detail, the cut-off values of ELISAs for P1, 2, 3, and 5 were 0.15, 0.155, 0.129, and 0.153, respectively. ELISAs for P1, P2, P3 and P5 detected 66.7, 70.0, 75.0, and 83.3% of *P. vivax* infection samples as vivax malaria positive, respectively (Table 1). In particular, ELISA with P5 showed best performance on detecting antibodies specific to *P. vivax* among the ELISAs with the peptides. This performance of antibody detection by PvLSA peptides is better than that by liver stage antigen of *P. falciparum* (70.7%) which has detected antibodies specific to *P. falciparum* in falciparum malaria patients in previous study [11]. In addition, this positive rate of the ELISA with P5 (83.3%) was higher compared to that of ELISA with CSP of *P. vivax* (11.26-21.01%), a promising vaccine candidate of vivax malaria, using samples from vivax malaria patients in ROK [23].

**Table 1:** Positive rate of vivax malaria by ELISAs with peptides (P1, 2, 3 and 5) spanning on PvLSA

**Peptides**	**P1**	**P2**	**P3**	**P5**
No. of positive sample (%)	40 (66.7)	42 (70.0)	45 (75.0)	50 (83.3)
No. of negative sample (%)	20 (33.3)	18 (30.0)	15 (25.0)	10 (16.7)

Although PvLSA and liver stage antigens of *P. falciparum* (PfLSA-1 and PfLSA-3) are antigenic proteins expressed by malaria parasites in hepatocytes, PvLSA and PfLSAs are distinct at the genomic sequence level. PvLSA is homologous with the liver stage antigen of *P. cynomolgi* (PCYB_092710, PlasmDB), but not with two liver stage antigens of *P. falciparum*. In addition, PvLSA does not have the specific repeat and non-repeat domains of the liver stage antigens of *P. falciparum*. Although PvLSA and the liver stage antigens of falciparum malaria (PfLSA-1 and PfLSA-3) have different functions in the liver stages of vivax and falciparum malaria.

Studies on molecules expressed by malarial parasites during the liver stage, such as, on liver stage antigen, could help identify the mechanisms responsible for the long-term survival of vivax parasites in the human liver and of latent infection. In addition, serodiagnostic methods and vaccines based on synthetic peptides have been recently developed for parasitic diseases and other pathogenic infections [16,24]. Therefore, we suggest further studies on the five peptides are warranted to provide more insight on the liver stage.

## Conclusions

We report for the first time, the characterization of a liver stage antigen of *P. vivax* using five synthesized peptides located on PvLSA. Specific anti-sera produced using the respective peptides were found to react with *P. vivax* parasites in human liver cells. Furthermore, peptides specifically reacted with sera from vivax malaria patients by ELISA. Based on these results, further studies would provide more insight on the liver stage of vivax malaria.

## Competing interests

The authors declare that they have no proprietary, commercial, or financial interests that could be construed to have inappropriately influenced this study. The authors declare that they have no competing interests.

## Authors’ contributions

JYK designed the study and revised the manuscript. YKG, EJS, YKC, HIS, and SYJ performed the experiments. YKG analyzed the data and drafted the manuscript. JS, CKC, SHC, and WJL helped design the study and revise the manuscript. All authors read and approved the final manuscript.

## Supplementary Material

Additional file 1: Figure S1 Alignment of six isolates of PvLSA and their SNP. Click here for file
