# Supplementary material for: First characterization of Plasmodium vivax liver stage antigen (PvLSA) using synthetic peptides
Source: Parasit Vectors. 2014 Feb 12;7:64. doi: 10.1186/1756-3305-7-64 (PMC3925417; doi:10.1186/1756-3305-7-64)

Supplementary figure 1. Alignment of six isolates of PvLSA and their SNP

: SNP

: Intron part

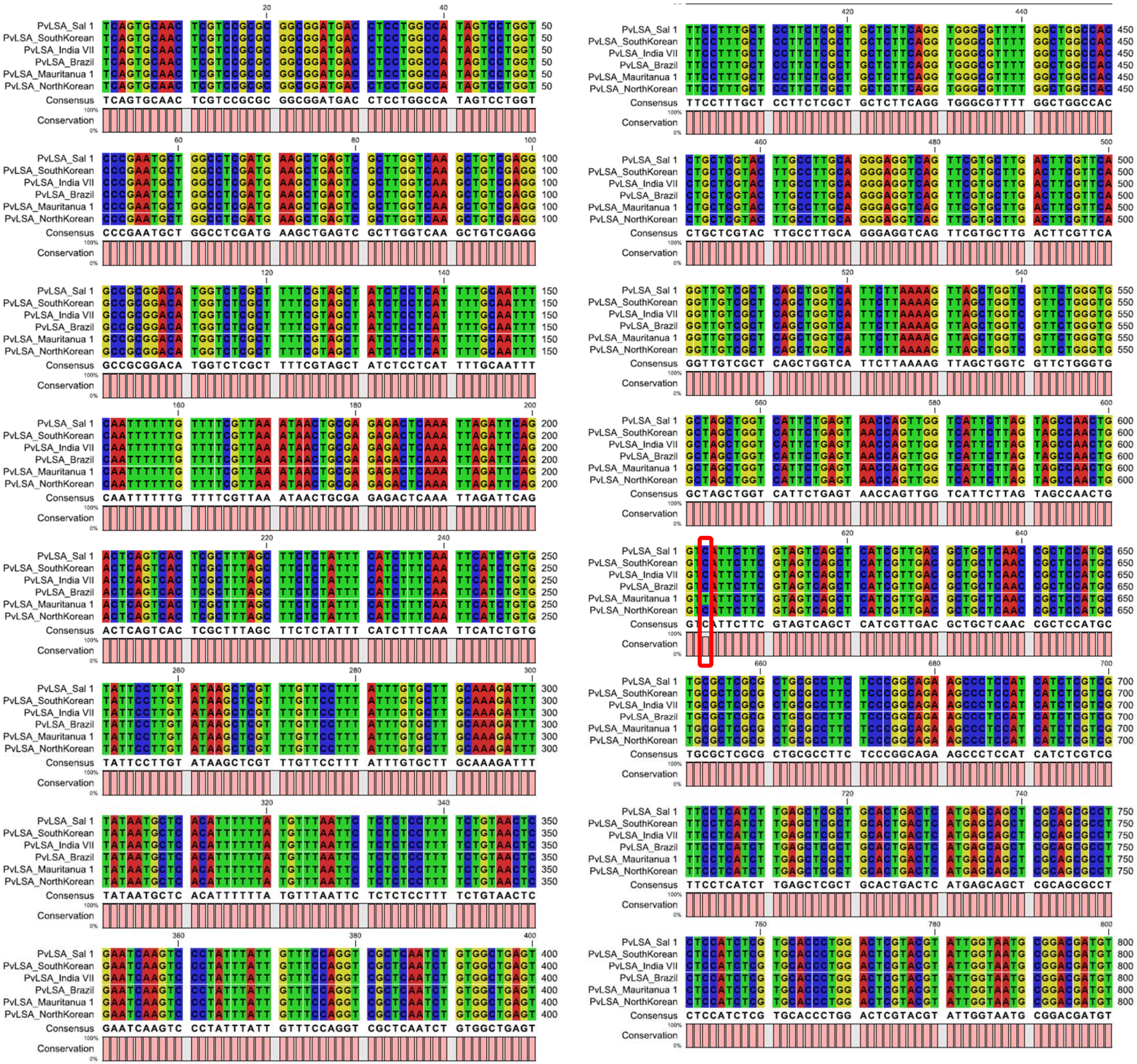

Supplementary figure 1. Alignment of six isolates of PvLSA and their SNP

: SNP

: Intron part

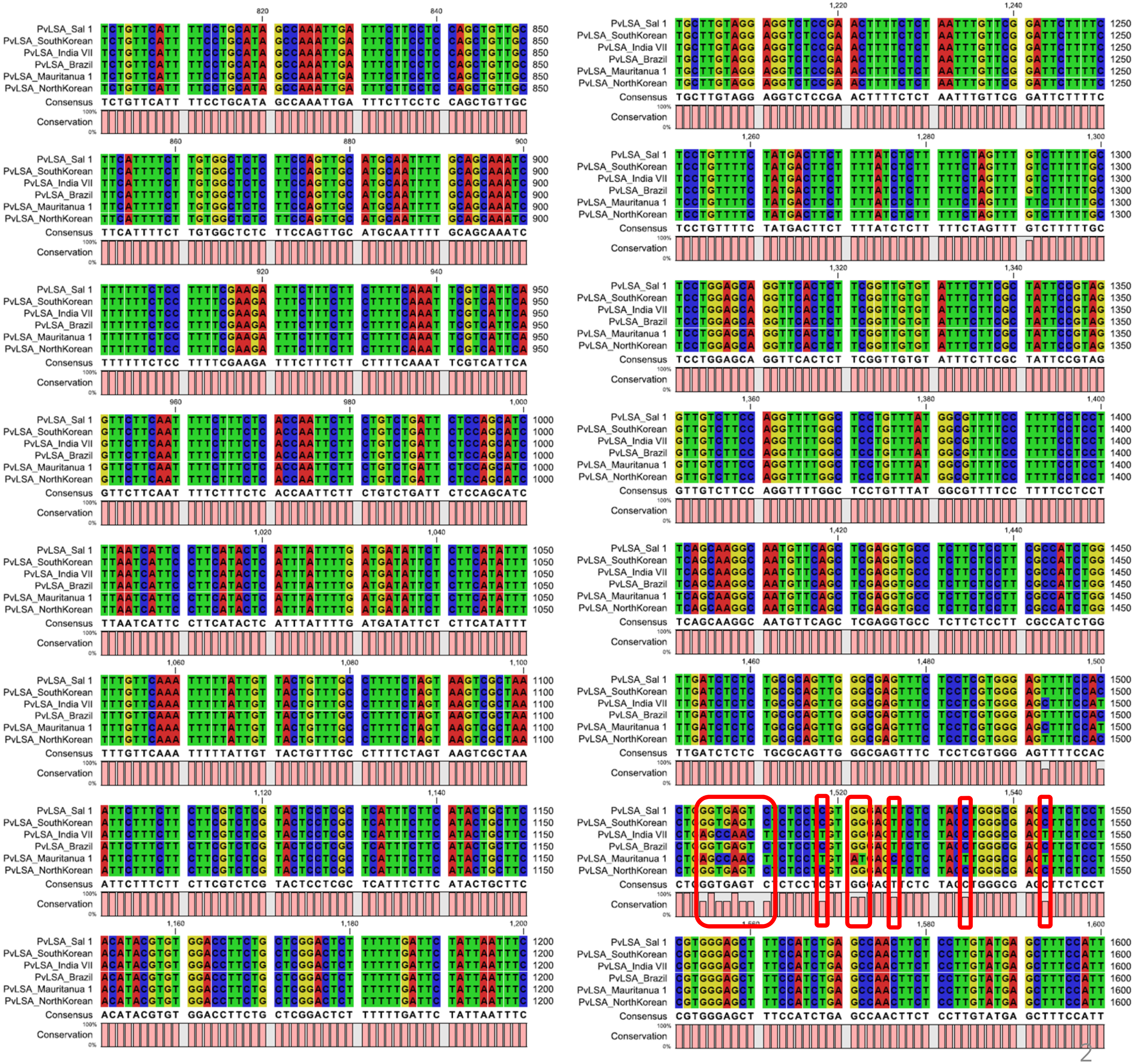

Supplementary figure 1. Alignment of six isolates of PvLSA and their SNP

: SNP

: Intron part

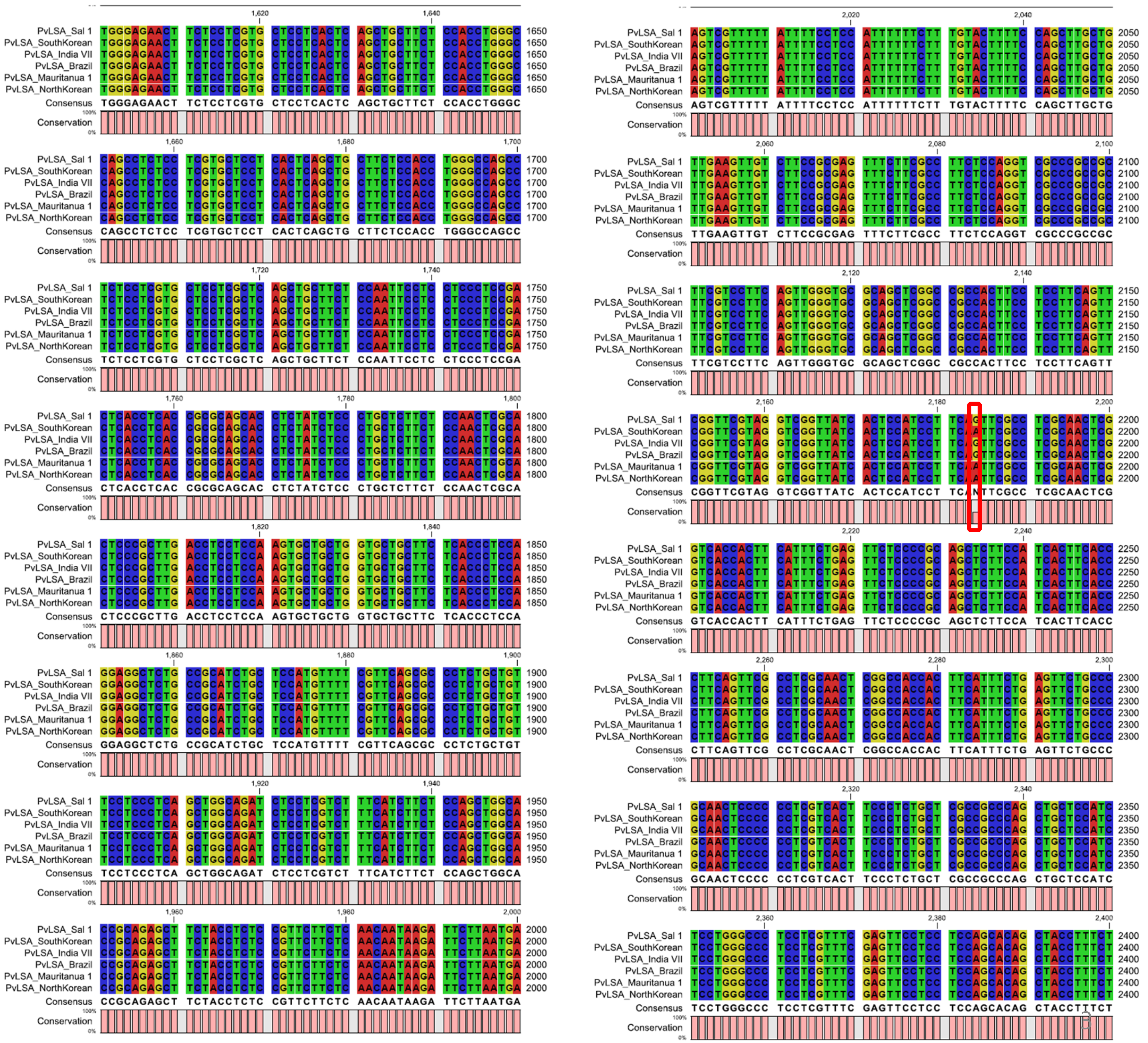

Supplementary figure 1. Alignment of six isolates of PvLSA and their SNP

: SNP

: Intron part

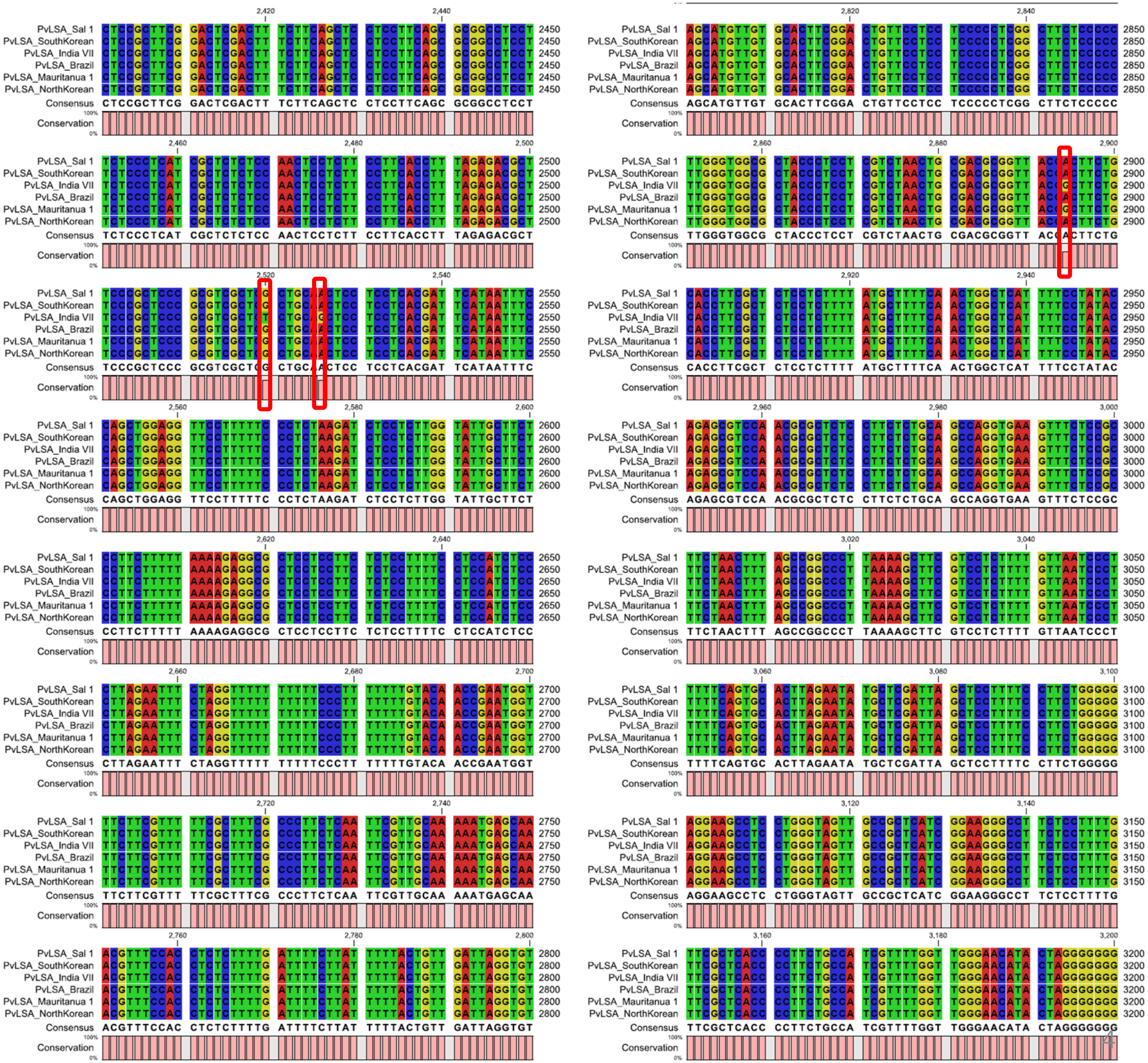

Supplementary figure 1. Alignment of six isolates of PvLSA and their SNP

: SNP

: Intron part

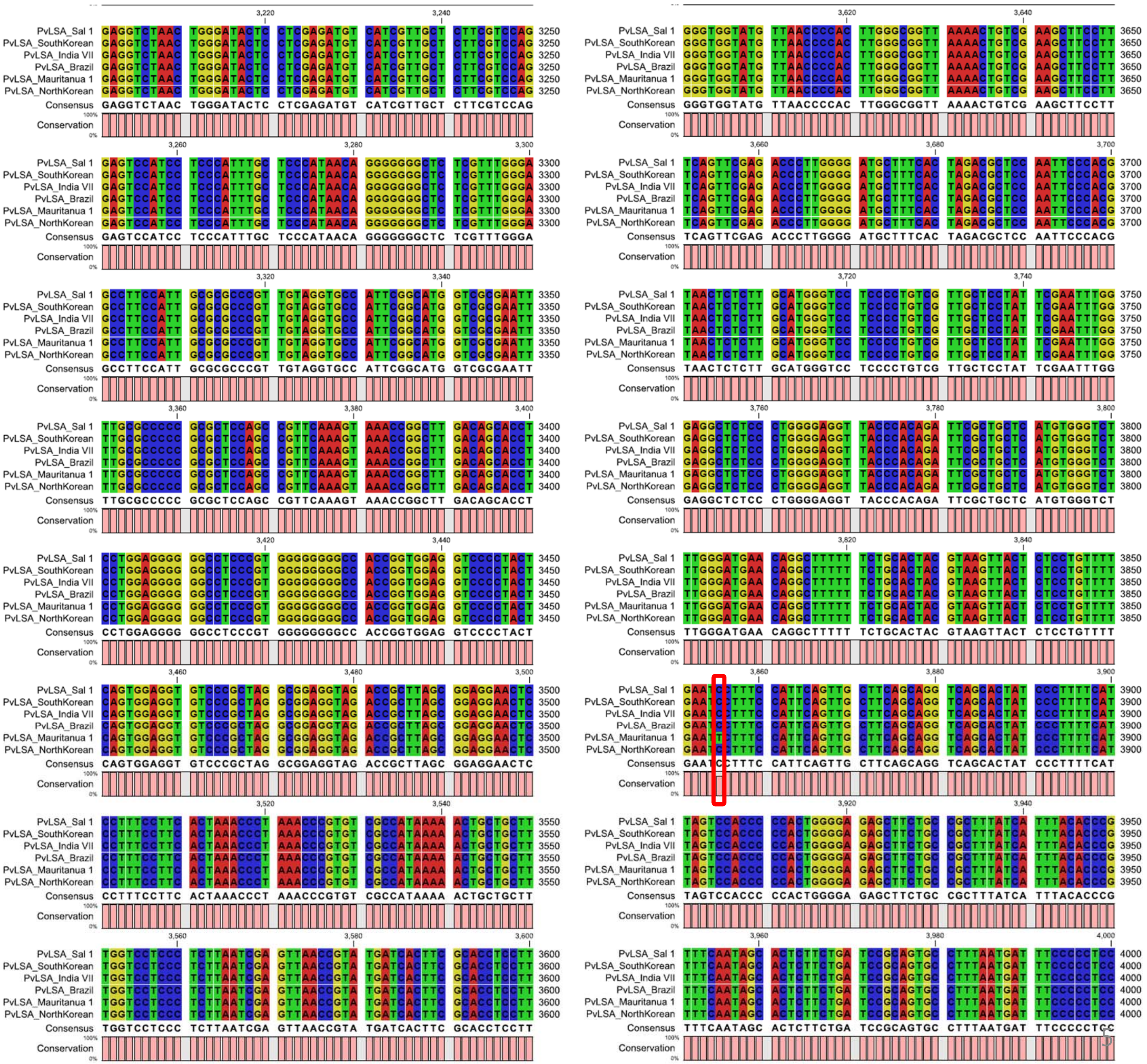

Supplementary figure 1. Alignment of six isolates of PvLSA and their SNP

: SNP

: Intron part

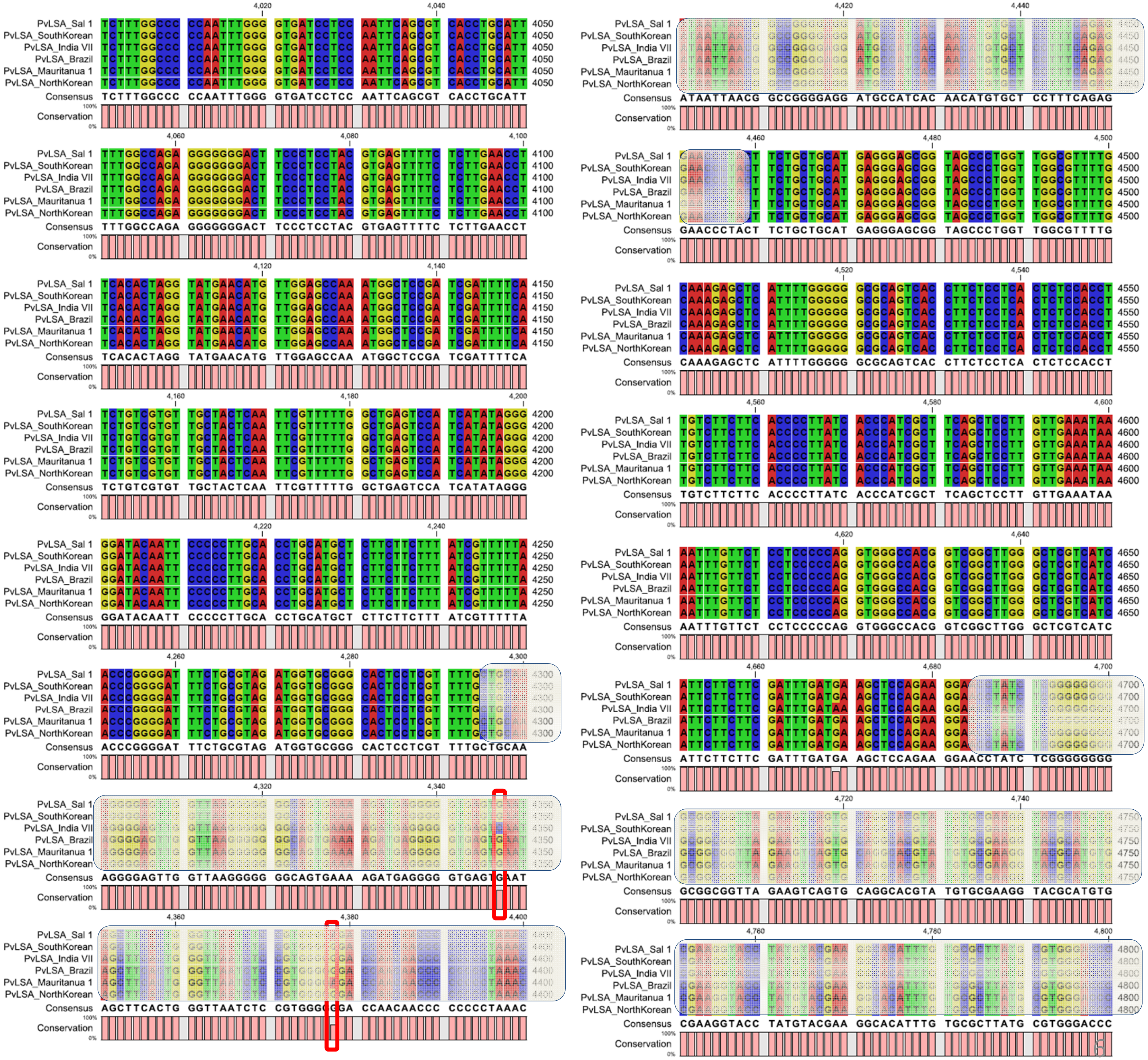

Supplementary figure 1. Alignment of six isolates of PvLSA and their SNP

: SNP

: Intron part

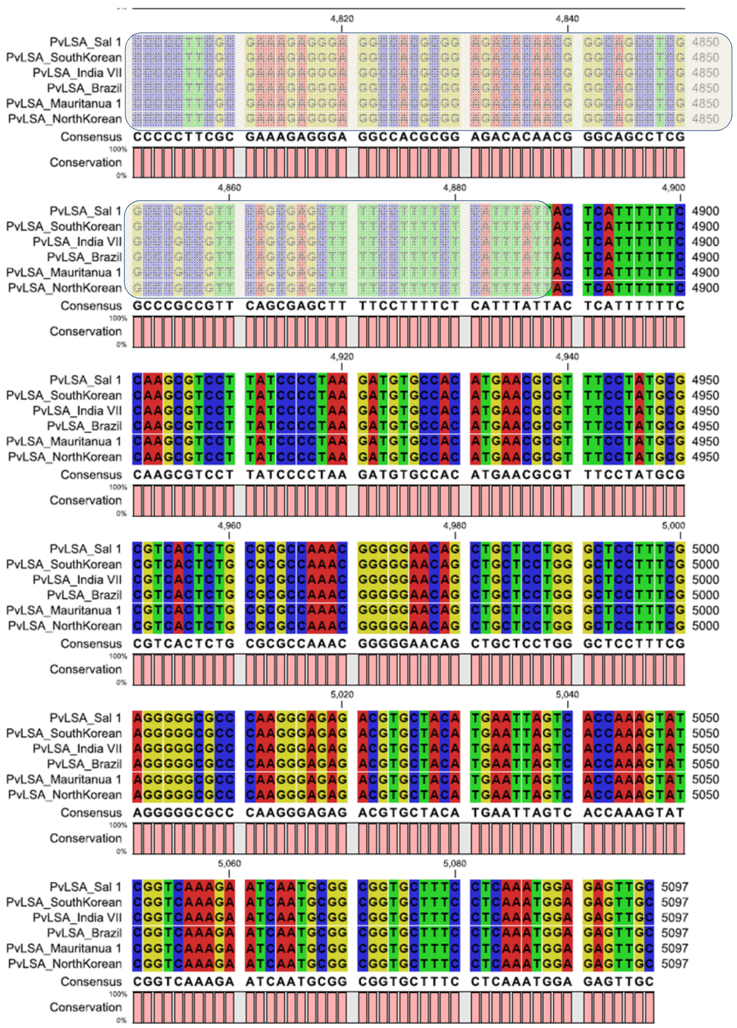

Supplement: Additional file 1: Figure S1 — Alignment of six isolates of PvLSA and their SNP. [file 1756-3305-7-64-S1.pdf]
